# Molecular residual disease detection in resected, muscle-invasive urothelial cancer with a tissue-based comprehensive genomic profiling–informed personalized monitoring assay

**DOI:** 10.3389/fonc.2023.1221718

**Published:** 2023-07-31

**Authors:** Thomas Powles, Amanda Young, Halla Nimeiri, Russell W. Madison, Alexander Fine, Daniel R. Zollinger, Yanmei Huang, Chang Xu, Ole V. Gjoerup, Vasily N. Aushev, Hsin-Ta Wu, Alexey Aleshin, Corey Carter, Nicole Davarpanah, Viraj Degaonkar, Pratyush Gupta, Sanjeev Mariathasan, Erica Schleifman, Zoe June Assaf, Geoffrey Oxnard, Priti S. Hegde

**Affiliations:** ^1^ Barts Experimental Cancer Medicine Centre, Barts Cancer Institute, Queen Mary University of London ECMC, Barts Health, London, United Kingdom; ^2^ Foundation Medicine, Cambridge, MA, United States; ^3^ Natera, Austin, TX, United States; ^4^ Roche/Genentech, South San Francisco, CA, United States

**Keywords:** MRD, CtDNA, bladder cancer, immunotherapy, comprehensive genomic profiling, next-generation sequencing, monitoring assay

## Abstract

**Introduction:**

Circulating tumor DNA (ctDNA) detection postoperatively may identify patients with urothelial cancer at a high risk of relapse. Pragmatic tools building off clinical tumor next-generation sequencing (NGS) platforms could have the potential to increase assay accessibility.

**Methods:**

We evaluated the widely available Foundation Medicine comprehensive genomic profiling (CGP) platform as a source of variants for tracking of ctDNA when analyzing residual samples from IMvigor010 (ClinicalTrials.gov identifier NCT02450331), a randomized adjuvant study comparing atezolizumab with observation after bladder cancer surgery. Current methods often involve germline sampling, which is not always feasible or practical. Rather than performing white blood cell sequencing to filter germline and clonal hematopoiesis (CH) variants, we applied a bioinformatic approach to select tumor (non-germline/CH) variants for molecular residual disease detection. Tissue-informed personalized multiplex polymerase chain reaction–NGS assay was used to detect ctDNA postsurgically (Natera).

**Results:**

Across 396 analyzed patients, prevalence of potentially actionable alterations was comparable with the expected prevalence in advanced disease (13% *FGFR2/3*, 20% *PIK3CA*, 13% *ERBB2*, and 37% with elevated tumor mutational burden ≥10 mutations/megabase). In the observation arm, 66 of the 184 (36%) ctDNA-positive patients had shorter disease-free survival [DFS; hazard ratio (HR) = 5.77; 95% confidence interval (CI), 3.84–8.67; *P* < 0.0001] and overall survival (OS; HR = 5.81; 95% CI, 3.41–9.91; *P* < 0.0001) compared with ctDNA-negative patients. ctDNA-positive patients had improved DFS and OS with atezolizumab compared with those in observation (DFS HR = 0.56; 95% CI, 0.38–0.83; *P* = 0.003; OS HR = 0.66; 95% CI, 0.42–1.05). Clinical sensitivity and specificity for detection of postsurgical recurrence were 58% (60/103) and 93% (75/81), respectively.

**Conclusion:**

We present a personalized ctDNA monitoring assay utilizing tissue-based FoundationOne^®^ CDx CGP, which is a pragmatic and potentially clinically scalable method that can detect low levels of residual ctDNA in patients with resected, muscle-invasive bladder cancer without germline sampling.

## 1 Introduction

Muscle-invasive bladder cancer (MIBC) is treated with radical cystectomy and pelvic lymphadenectomy, but a considerable proportion of patients experience disease recurrence, with recurrence-free survival at 5 and 10 years of 68% and 66%, respectively (1, 2). Standard computed tomography is useful to detect relapse and monitor response, but its monitoring potential is restricted by detection limits and variability of measurements (3). Early detection of molecular residual disease (MRD) could allow identification of relapse ahead of imaging, which may help in the selection of patients suitable for adjuvant therapy.

Historically, identifying patients with residual disease postsurgery versus those who attain cure from surgery has been a challenge. There is an unmet need for biomarkers to optimally select patients for adjuvant treatment after curative-intent surgery. Detection of postsurgical circulating tumor DNA (ctDNA) is now established to detect MRD and predict risk of recurrence in patients with high sensitivity, across various early-stage cancers, including bladder, breast, lung, colorectal, and colon cancers (3–8).

For bladder cancer, there is compelling rationale that the detection of MRD after curative-intent surgery may identify patients at a high risk of relapse who may benefit from adjuvant therapy (3, 6). Recent data from IMvigor010 (6) indicated that patients with MIBC who tested positive for ctDNA selected from tissue whole-exome sequencing (WES) at baseline had improvements in disease-free survival (DFS) and overall survival (OS) with adjuvant atezolizumab compared with those in observation. A link between tissue-based immune biomarkers and ctDNA positivity was also demonstrated (6).

A challenge with ctDNA detection is distinguishing tumor-derived variants from other sources [e.g., clonal hematopoiesis (CH)–derived mutations, germline mutations, and sequencing artifacts] (9). Current MRD methods based on WES incorporate sampling of the germline through peripheral blood mononuclear cells, but this adds complexity and is not always feasible or clinically practical. Here, building off a clinically available tumor next-generation sequencing platform, we use a bioinformatic approach to filter out germline and CH-derived mutations in the absence of matched normal tissue samples using tumor tissue samples from the IMvigor010 trial. Basing MRD detection off a globally scalable tissue platform could increase access to the emerging MRD paradigm while also creating an opportunity to leverage comprehensive genomic profiling (CGP) of resected disease to guide precision adjuvant therapy approaches (10).

## 2 Materials and methods

### 2.1 Tissue CGP to identify patient-specific alterations

**Figure 1:**
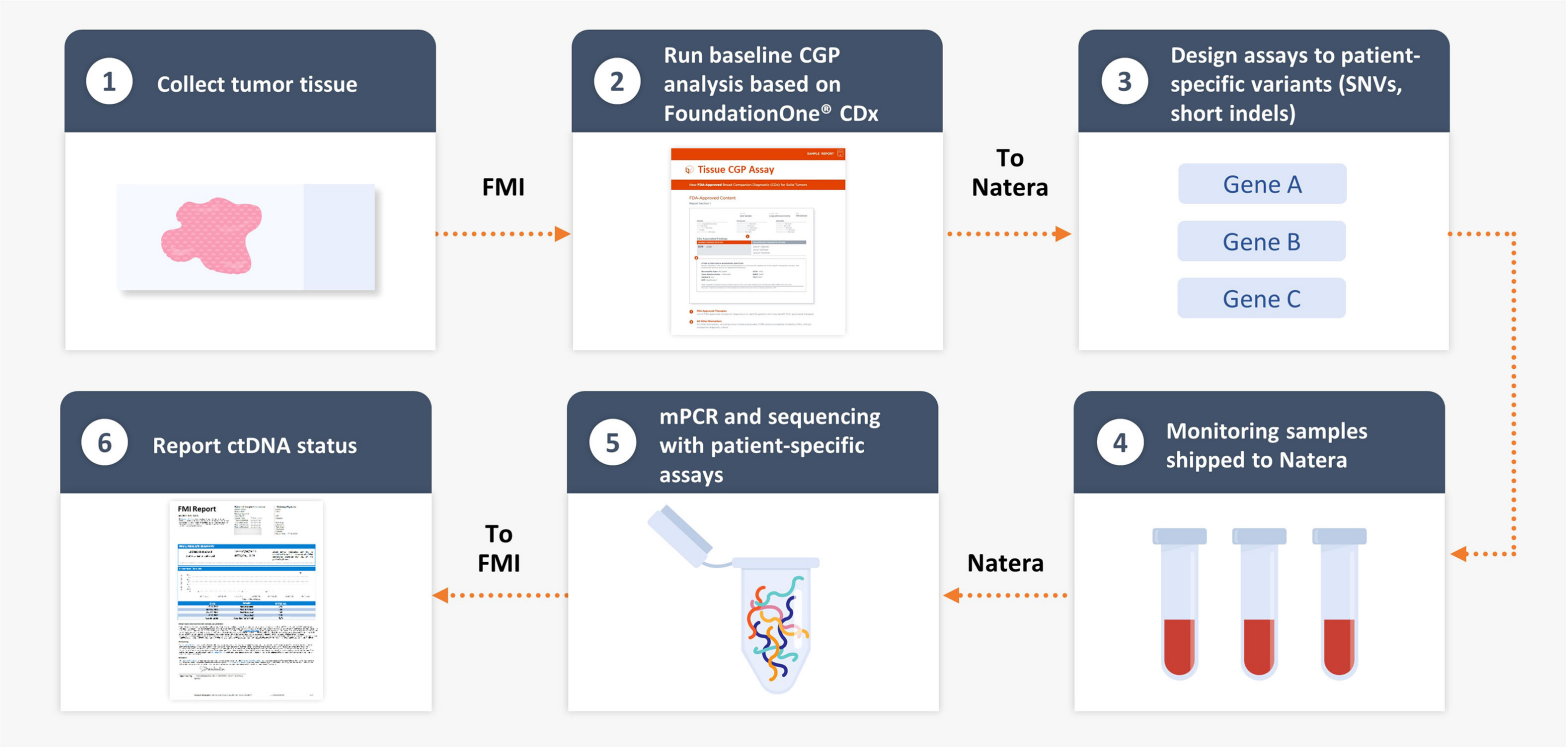
FoundationOne^®^ Tracker, a tissue-informed personalized ctDNA monitoring assay. CGP, comprehensive genomic profiling; ctDNA, circulating tumor DNA; FMI, Foundation Medicine, Inc.; mPCR, multiplex polymerase chain reaction; SNV, single-nucleotide variant.

Using genomic DNA from resected tumor tissue, CGP was performed retrospectively as described by Milbury et al. (11) using a clinical trial assay version of FoundationOne^®^ CDx to identify patient-specific alterations in the ctDNA cohort with the FoundationOne^®^ Tracker assay (**Figure 1**). This assay leverages a tissue-informed personalized ctDNA monitoring approach for evaluating therapeutic response and detecting molecular response for patients across tumor types. CGP was performed on patient samples in a Clinical Laboratory Improvement Amendments–certified, College of American Pathologists–accredited, New York State–approved laboratory (Foundation Medicine, Cambridge, MA). Approval for this study, including a waiver of informed consent and a Health Insurance Portability and Accountability Act of 1996 waiver of authorization, was obtained from the Western Institutional Review Board (protocol no. 20152817). Briefly, genomic DNA was extracted from formalin-fixed paraffin-embedded samples, and DNA was end-repaired and A-tailed, and adapters were ligated and hybridization-based captured, followed by sequencing on the Illumina^®^ HiSeq 4000 (12). Tumor mutational burden (TMB) was calculated from 0.8 megabase (Mb) of sequenced DNA, excluding driver and germline alterations (13). A novel, proprietary algorithm was used to predict somatic probability to minimize the selection of non-tumor–derived variants (germline, CH-derived, and sequencing artifacts). This algorithm includes a novel logistic regression model to predict probability of a variant being somatic (somatic probability score) based on the difference between the observed variant allele frequency (VAF) and the inferred expected germline VAF. This algorithm directly infers the expected germline allele frequency from known germline single-nucleotide polymorphisms located on adjacent genomic regions expected to have the same copy number with the variant in question. The algorithm then filters variants based on the somatic probability score, allele frequency and annotation, and comparison with databases of known single-nucleotide polymorphisms and CH variants. Short variants (SVs) with high somatic probability were selected and submitted for primer design for the monitoring assay.

### 2.2 Plasma multiplex polymerase chain reaction sequencing workflow

Multiplex polymerase chain reaction (PCR) sequencing workflow has been described previously (4, 6, 7). Briefly, the personalized amplicon design pipeline was used to design multiplex PCR primer pairs for each variant derived from the CGP result for each patient. The approach can select coding non-silent alterations in cancer-associated genes [termed pathogenic for alterations with known or likely oncogenic significance or termed variant of unknown significance (VUS) coding for alterations with unknown significance] as well as intronic or synonymous alterations (termed non-coding and synonymous) for monitoring (4–7). Predicted amplicons were ranked, and 2–16 were selected for each custom patient-specific panel. Blood sample processing, cell-free DNA library construction, and sequencing were described previously (6). VAFs were determined for each of the targeted variants. Samples with ≥2 variants above the detected threshold were defined as ctDNA-positive. Absolute ctDNA levels [reported as mean tumor molecules of plasma per milliliter (MTM/mL)] in the plasma were determined by normalizing VAF to the plasma volume and input cell-free DNA for each sample. All passing targets were used in the MTM/mL calculation, including those that were undetected.

### 2.3 The IMvigor010 study design

The IMvigor010 trial (NCT02450331) was a global, phase 3, open-label, randomized, and multicenter study that evaluated the efficacy and safety of adjuvant atezolizumab (up to 1 year or until recurrence of urothelial carcinoma or unacceptable toxicity) compared with observation in patients with muscle-invasive urothelial carcinoma who are at a high risk for recurrence after surgical resection. Imaging assessments for disease recurrence were performed at baseline and every 12 weeks for 3 years and every 24 weeks for years 4–5 and at year 6 (14). Details of the study have been published previously (6, 14). As study specimens have been analyzed previously (6), we investigated residual samples when available. Because residual samples were analyzed *post-hoc* with variable sample availability, a comparative analysis with the previously published MRD study was not undertaken as part of this initial analysis.

DFS was the primary endpoint of IMvigor010 defined by local (pelvic) or urinary tract recurrence, distant urothelial carcinoma metastasis, or death from any cause (14). The secondary efficacy endpoint was OS, defined as the time from randomization to death from any cause (14). The study was performed according to the Good Clinical Practice and the Declaration of Helsinki. Protocol approval was obtained from independent review boards or ethics committees at each site. An informed written consent was obtained from all patients.

### 2.4 Statistical analysis

This work was not part of the original statistical analysis plan from IMvigor010; therefore, the work is exploratory. The analysis includes the prognostic value of ctDNA positivity and the effect of atezolizumab on DFS and OS compared with placebo. Nominal *P*-values are given as an indicator of probability but do not represent statistical significance. Hazard ratios (HRs) for recurrence or death were estimated using a univariable Cox proportional hazards model. Survival distributions for DFS and OS were estimated using a Kaplan–Meier method and tested for difference between patient groups using the log-rank test.

A multivariable Cox proportional hazards model was used to estimate the HR between ctDNA-positive and ctDNA-negative patients in the observation arm adjusting for key clinical variables. A series of univariable Cox proportional hazards models with ctDNA status, nodal status, programmed cell death-ligand 1 (PD-L1) status, tumor stage, prior neoadjuvant chemotherapy, and number of lymph nodes were evaluated one at a time, followed by a multivariable Cox proportional hazards model, including only the variables that were statistically significant (*P* < 0.05) in the univariable models. Descriptive statistics were used to summarize clinical characteristics. All statistical analyses were performed in R version 4.0.2 (https://www.R-project.org).

For CGP analysis, the frequency of genomic alterations was assessed from metastatic bladder specimens in the FoundationCORE^™^ database. To select a cohort representative of metastatic disease, specimens that were collected from the bladder, surrounding tissue (ureter, pelvis, urethra, penis, iliac crest, vulva, and anus), soft tissue, or lymph nodes were excluded. Comparisons of genomic alteration frequency between the IMvigor010 cohort and the FoundationCORE^™^ cohort were made with Fisher exact tests adjusted for multiple comparisons using the Benjamini–Hochberg procedure. Homologous recombination deficiency was defined by pathogenic mutations in any of the genes *BRCA1*, *BRCA2*, *ATM*, *BARD1*, *BRIP1*, *CDK12*, *CHEK1*, *CHEK2*, *FANCL*, *PALB2*, *RAD51B*, *RAD51C*, *RAD51D*, and *RAD54L.*

## 3 Results

### 3.1 Characteristics of the clinical cohort

**Figure 2:**
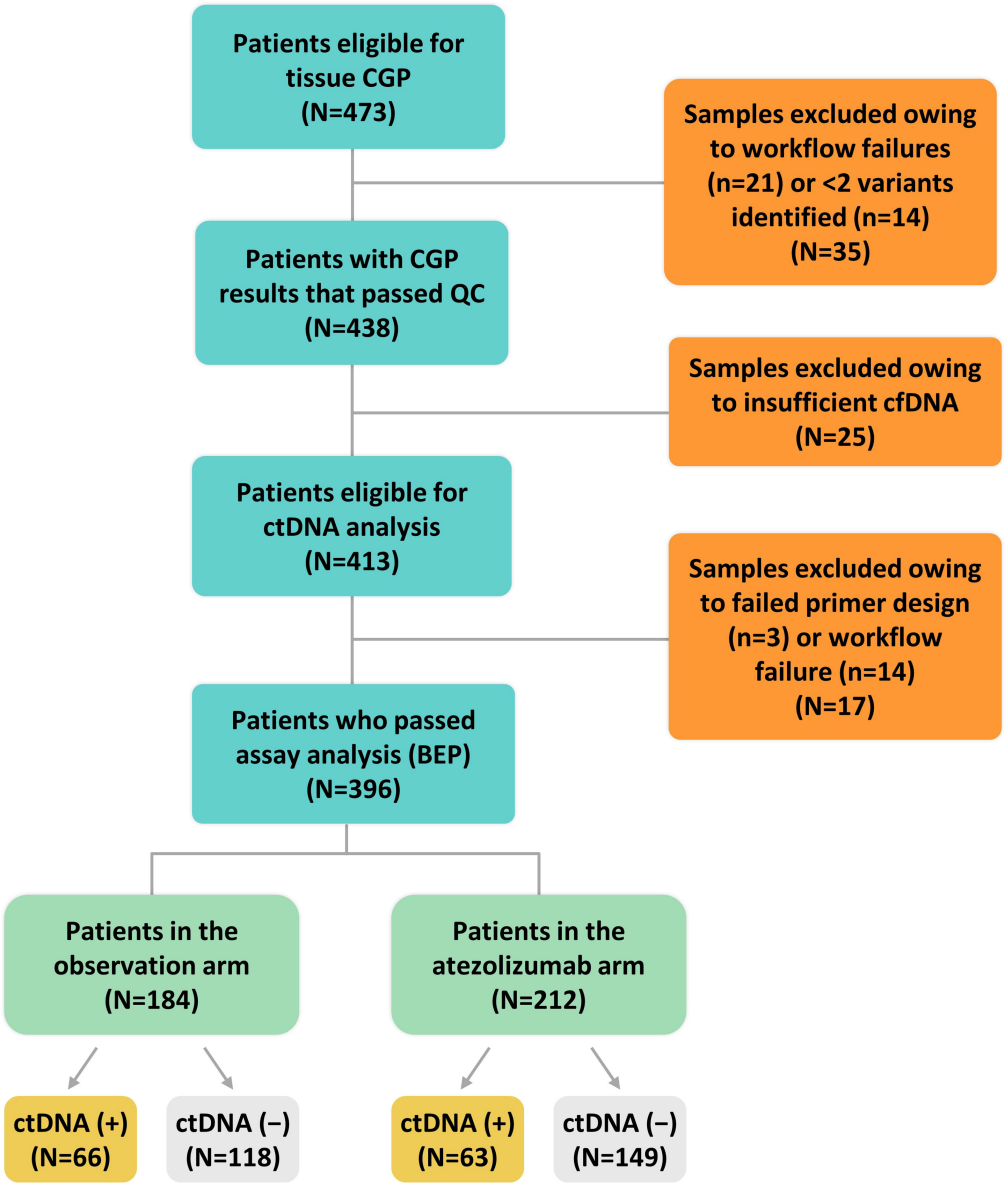
CONSORT diagram. BEP, biomarker-evaluable population; cfDNA, cell-free DNA; CGP, comprehensive genomic profiling; ctDNA, circulating tumor DNA; QC, quality control.

We evaluated a personalized MRD assessment and recurrence-monitoring assay based on tissue-informed CGP, which uses a computational algorithm to filter out non-tumor–derived variants, thus avoiding the germline sampling step that is associated with WES methods. The tumor-derived variants were used to measure plasma-based ctDNA levels in a personalized assay at a single MRD time point with a median of 11 weeks postsurgery (**Figure 1**) and with a median follow-up of 28 months. In the IMvigor010 trial, 809 patients were enrolled as follows: 403 in the observation arm and 406 in the atezolizumab arm [intention-to-treat (ITT) (15) population]. There were 473 patients eligible for CGP (58% of the ITT population), and, ultimately, 396 of these 473 patients were included in the biomarker-evaluable population (BEP) as follows: 184 patients in the observation arm and 212 patients in the atezolizumab arm (**Figure 2**). Baseline clinical characteristics of the BEP were well balanced between arms and similar to the ITT population (**Supplementary Table 1**). At the postsurgical time point, the ctDNA-positive population included 66 patients in the observation arm [(66/184 (36%)] and 63 patients in the atezolizumab arm [63/212 (30%)]. Survival outcomes were similar between the ITT population and the BEP (**Supplementary Figure 1**).

### 3.2 CGP at baseline identifies potentially actionable alterations and high TMB in patients with bladder cancer

**Figure 3:**
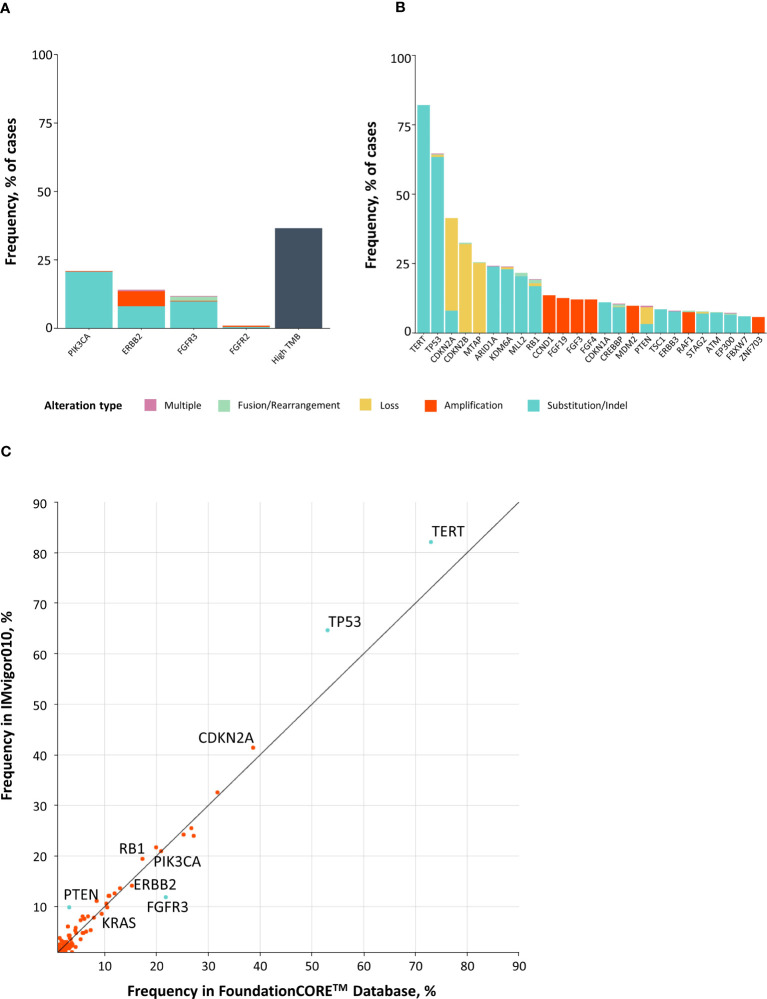
Comprehensive genomic profiling at baseline identifies potentially actionable alterations in patients with bladder cancer. **(A, B)** Top 40 most commonly altered genes in IMvigor010, including potentially actionable alterations and high TMB **(A)**, as well as other genomic alterations **(B)**. **(C)** X–Y plot comparing prevalence of genomic alterations in MIBC from IMvigor010 versus advanced-stage bladder cancer from the FoundationCORE^™^ database. MIBC, muscle-invasive bladder cancer; TMB, tumor mutational burden.

CGP analysis of 396 patients with MIBC across both arms revealed potentially actionable alterations, including *FGFR2/3* SVs and fusions (13%), *ERBB2* SVs and amplifications (13%), and *PIK3CA* SVs (20%) (**Figures 3A, B**). TMB high (≥10 mutations/Mb) was identified in 37% (145/392) of patients. Among the 145 TMB high patients, only five were microsatellite instability high, three had unknown status, and 137 were microsatellite stable. Comparing CGP results with a real-world cohort with metastatic bladder cancer (16, 17), a similar distribution was seen, although a few substantial differences were found for *PTEN* [odds ratio (OR) = 0.30], *FGFR3* (OR = 2.07), *TP53* (OR = 0.62), and *TERT* (OR = 0.62) (*P* < 0.05 for all) (**Figure 3C**). Together, this analysis demonstrates that patients with MIBC harbor multiple potentially actionable alterations in *FGFR2/3*, *ERBB2*, and *PIK3CA* and frequently exhibit high TMB.

### 3.3 Characterization of monitorable variant types and their distribution in patient cohorts

**Figure 4:**
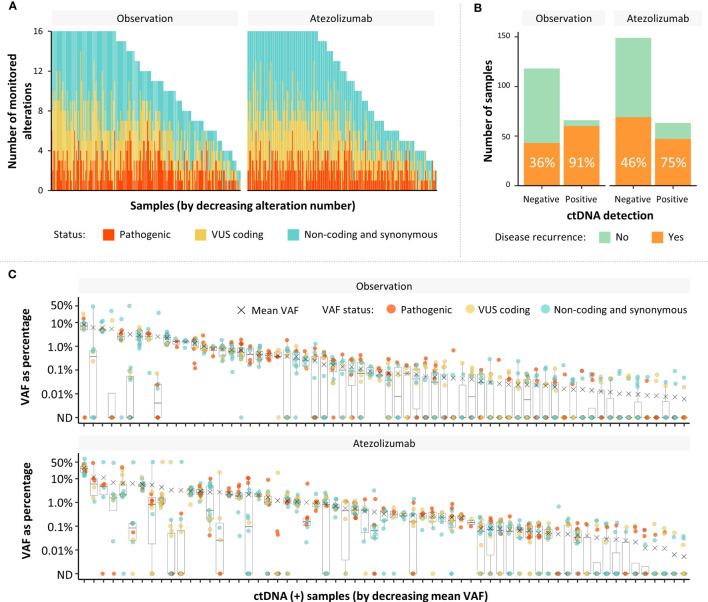
Characterization of monitorable variant types, as well as their distribution in patient cohorts. **(A)** Distribution and number of monitorable variants in patients in the observation and atezolizumab arms of IMvigor010 (pathogenic and VUS coding are coding cancer-associated alterations; non-coding and synonymous are intronic or synonymous alterations). **(B)** Percentage of patients with disease progression in ctDNA populations by arm in the IMvigor010 study. **(C)** VAF distribution of each monitored alteration in ctDNA-positive samples by arm. ctDNA, circulating tumor DNA; ND, not detected; VAF, variant allele frequency.

To maximize the number of monitorable alterations, the assay included coding cancer-associated alterations (termed pathogenic or VUS coding) and intronic or synonymous alterations (termed non-coding and synonymous) for tracking of 2–16 variants using a personalized assay (4–7). The distribution and the number of monitorable variants were comparable between patients in the observation and atezolizumab arms of IMvigor010 (**Figure 4A**). The median number of monitorable variants was 12 in each arm. As expected, most ctDNA-positive patients experienced disease progression, which was more pronounced in the observation arm (**Figure 4B**). Of the 129 ctDNA-positive patients, 107 (82.9%) experienced disease progression, which included 60/66 (90.9%) in the observation arm and 47/63 (74.6%) in the atezolizumab arm. The plasma VAF distribution of the different tracked variant statuses (mean VAF by status: non-coding and synonymous = 2.53%, pathogenic = 2.11%, and VUS coding = 1.92%, difference not significant by a Kruskal–Wallis test) was similar across all samples (**Figure 4C**), and the distribution of MTM/mL values was similar between observation and atezolizumab arms (**Supplementary Figure 2**). Alterations across a wide range of VAFs (range, 0.01%–68.6%; median = 0.3%) were detected in ctDNA-positive samples, and monitorable variant statuses were similarly distributed across all samples, exhibiting no obvious bias. In sum, ctDNA-positive samples were readily identified across a range of patients with a differing number of variants designed, VAF, alteration status, and treatment arms.

### 3.4 Association of ctDNA status with patient outcomes confirms ctDNA as a prognostic and predictive biomarker

**Figure 5:**
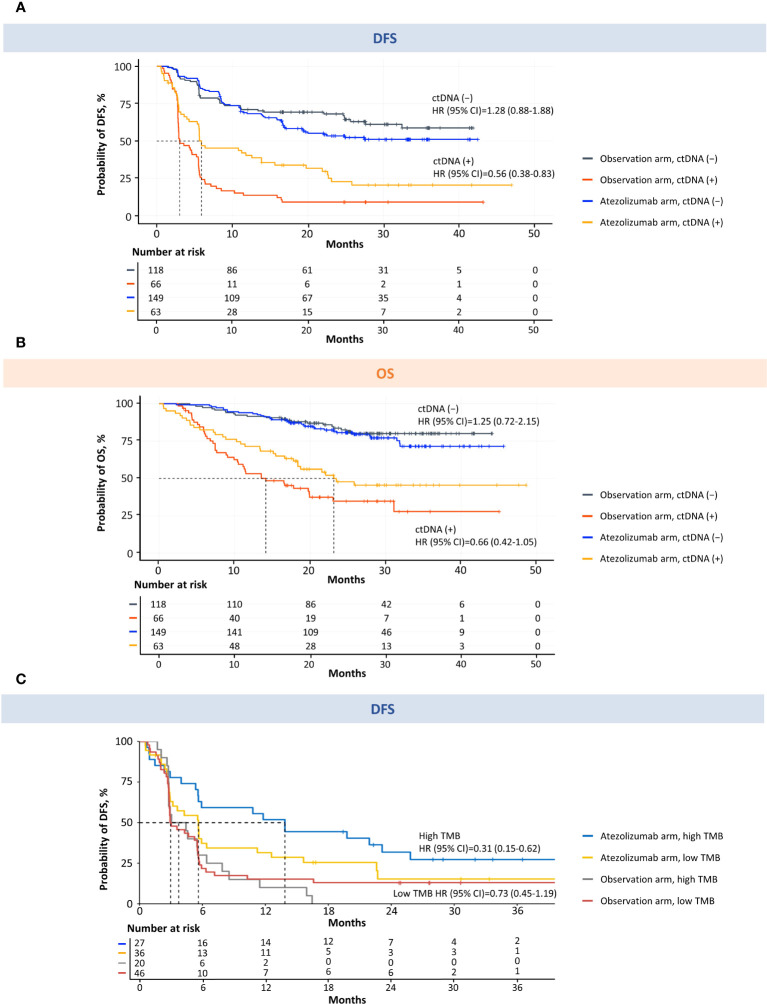
MRD detection is prognostic for DFS and OS and predictive of atezolizumab response in patients with MIBC. Kaplan–Meier estimates of DFS **(A)** and OS **(B)** for patients monitored and stratified by ctDNA detection (MRD) at the postsurgical time point (univariable analysis). Kaplan–Meier estimates of DFS for ctDNA-positive patients monitored and stratified by TMB **(C)**. High TMB was defined as ≥10 mutations/Mb. Low TMB was defined as <10 mutations/Mb. CI, confidence interval; ctDNA, circulating tumor DNA; DFS, disease-free survival; Mb, megabase; MIBC, muscle-invasive bladder cancer; MRD, molecular residual disease; OS, overall survival; TMB, tumor mutational burden.

Analysis of DFS in the observation arm showed that the ctDNA-positive patients had a higher risk of disease recurrence than the ctDNA-negative patients (**Figure 5A**; **Supplementary Table 2**). The median DFS was 3.0 months (95% CI, 2.9–5.5) in the ctDNA-positive population and was not reached in the ctDNA-negative population (observation arm DFS HR = 5.77; 95% CI, 3.84–8.67; *P* < 0.0001). Of the 66 ctDNA-positive patients in the observation arm, 60 had relapsed at time of analysis; therefore, the positive predictive value was 91%. Of the 118 ctDNA-negative patients in the observation arm, 75 had not relapsed at time of analysis, resulting in a negative predictive value of 64%. In this study, the clinical sensitivity of this ctDNA assay to detect radiologic relapse, corresponding to the true positive rate, was 58% (60/103 in the observation arm) and the specificity was 93% (75/81 in the observation arm). Analysis of OS in the observation arm showed that the ctDNA-positive patients had a higher risk of death than the ctDNA-negative patients (**Figure 5A**; **Supplementary Table 2**). The median OS was 14.1 months (95% CI, 10.5–23.0) in the ctDNA-positive population and was not reached in the ctDNA-negative population (observation arm OS HR = 5.81; 95% CI, 3.41–9.91; *P* < 0.0001).

Furthermore, patients who were positive for ctDNA had improved DFS and OS with adjuvant atezolizumab compared with patients undergoing observation (**Figures 5A, B**; **Supplementary Table 2**). The median DFS was 5.9 months in ctDNA-positive patients who received adjuvant atezolizumab versus 3.0 months in ctDNA-positive patients who underwent observation (HR = 0.56; 95% CI, 0.38–0.83; *P* = 0.003) (**Figure 5A**; **Supplementary Table 2**). The median OS was 23.1 months in ctDNA-positive patients who received adjuvant atezolizumab versus 14.1 months in ctDNA-positive patients who underwent observation (HR = 0.66; 95% CI, 0.42–1.05; *P* = 0.081) (**Figure 5B**; **Supplementary Table 2**). When the ctDNA-positive population was stratified by high TMB (≥10 mutations/Mb) versus low TMB (<10 mutations/Mb), the direction, magnitude, and strength of associations with DFS and OS were in line with what would be anticipated based upon the cohort powering (**Figure 5C**, **Supplementary Figures 3, 4**; **Supplementary Table 3**), and prior TMB assay performance in other bladder cancer cohorts (18–20). In addition, we found that stratifying the ctDNA-positive population by PD-L1 status demonstrated a benefit in DFS and OS for high versus low PD-L1 in the atezolizumab arm (**Supplementary Figures 5, 6**). We performed an exploratory biomarker analysis of *FGFR3*, *ERBB2*, *PIK3CA*, *CDKN2A* alterations, *MDM2* amplification, and homologous recombination deficiency in the ctDNA-positive population comparing atezolizumab with the observation arm, which suggested that *FGFR3* wild-type, but not pathogenic mutants, favored atezolizumab, consistent with the JAVELIN 100 analysis (19) (**Supplementary Figure 7**, univariable analysis; **Supplementary Figure 8**, multivariable analysis). No significant difference in DFS or OS was found between arms for ctDNA-negative patients (DFS HR = 1.28; 95% CI, 0.88–1.88; *P* = 0.195; OS HR = 1.25; 95% CI, 0.72–2.15; *P* = 0.432) (**Figures 5A, B**; **Supplementary Table 2**).

**Figure 6:**
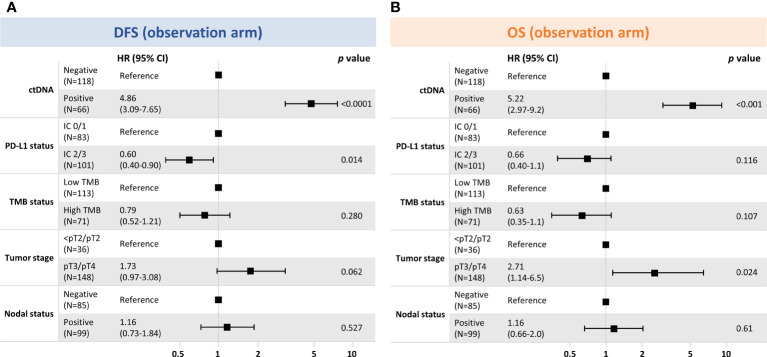
Multivariable exploratory analyses^a^ of clinical factors associated with **(A)** DFS and **(B)** OS in the observation arm. **(A)** DFS where number of events were 103, global *P*-value (log rank), 2.46e^–16^; AIC, 920.88; concordance index, 0.76. **(B)** OS where number of events were 62; global *P*-value (log rank), 3.60e^−11^; AIC, 564.55; concordance index, 0.75. ^a^Model includes variables with *P* < 0.05 in univariable models. AIC, Akaike information criterion; CI, confidence interval; ctDNA, circulating tumor DNA; DFS, disease-free survival; IC, immune cell; PD-L1, programmed cell death-ligand 1; pT, primary tumor; OS, overall survival; TMB, tumor mutational burden.

To determine which prognostic factors were the most significantly associated with DFS and OS, we performed univariable (**Supplementary Figure 9**) and multivariable exploratory analyses (**Figure 6**). These analyses confirmed that ctDNA status independently identified patients with improved outcomes in the observation arm and, of the clinical variables, was the strongest predictor of both DFS and OS.

## 4 Discussion

This study shows that a tissue-informed personalized ctDNA monitoring assay, built off a CGP panel, could identify postsurgical bladder patients at a high risk of recurrence and that ctDNA-positive patients had improved outcomes with atezolizumab versus observation. Although treatment benefit in ctDNA-positive patients had been previously demonstrated for IMvigor010 (6), the novelty in our approach lies in the use of a globally available CGP assay as tissue baseline to inform the design of the assay. Unlike WES-based assays, this approach does not require germline sequencing from matched normal samples because an enhanced algorithm effectively filters out non-tumor variants. Furthermore, this approach represents an adaptation of the baseline CGP to include not only pathogenic and VUS coding but also non-coding and synonymous variants for monitoring, which maximizes the number of trackable variants and sensitivity. This novel approach demonstrated the ability of ctDNA to be prognostic by accurately identifying, even at one time point, the MRD-positive population at increased risk of relapse, and ctDNA status was the only predictor of DFS and OS in the multivariable analysis (*P* < 0.001) (**Figure 6**). We are hopeful that this approach, which was recently granted breakthrough device designation by the United States Food and Drug Administration (21), could represent a novel and convenient approach for MRD detection that uses a well-validated tissue CGP baseline assay both to reveal potentially actionable alterations and enable personalized ctDNA monitoring.

Here, we report on CGP in MIBC, identifying genomic alterations and complex biomarkers. Utilizing an actionable tissue baseline derived from a pragmatic, widely available, scalable, and extensively validated tissue CGP assay could allow for identification of potentially actionable alterations at baseline and the potential for a tailored approach to adjuvant-targeted therapies. We confirm previous findings using a different method that a combination of ctDNA and tissue-based biomarkers (TMB/PD-L1) will help patient selection. While our data were underpowered to show this point and the work was exploratory, trends toward benefit by combining biomarkers were apparent.

Potentially actionable genomic alterations included *FGFR2/3*, *ERBB2*, and *PIK3CA*, and alteration frequencies of these genes in resected MIBC from IMvigor010 were similar to the prevalence in advanced-stage urothelial carcinoma based on the FoundationCORE^™^ dataset. In addition, the prevalence of the top genomic alterations in urothelial carcinoma that we observed was similar to previous reports (15, 22, 23). Although fibroblast growth factor receptor inhibitors, including the United States Food and Drug Administration–approved erdafitinib, are listed in the National Comprehensive Cancer Network guidelines for advanced or metastatic bladder cancer, targeting strategies for HER2 and PIK3CA involve investigational agents in a clinical trial setting (24, 25). Adding utility, we showed in an exploratory biomarker analysis of the ctDNA-positive treatment arm that patients with *FGFR3* pathogenic mutations might not benefit from atezolizumab, consistent with previous results (19), but the cohort size was limiting. There is additional utility in using liquid biopsy CGP to detect potentially actionable alterations in *FGFR3* or other genes that may be acquired later in disease progression in subsets of metastatic samples. While TMB has not been formally tested as a biomarker in randomized trials, exploratory analysis has highlighted it as a potential biomarker (18–20, 26). Recent real-world evidence also indicates that TMB can be a predictive biomarker for immune checkpoint inhibitor versus chemotherapy benefit in urothelial carcinomas (27). Our data, while exploratory and underpowered, also show a trend toward benefit in this population.

Limitations of this study include that only one time point was assessed, and some tumors may not shed ctDNA after radical cystectomy. Therefore, detecting low ctDNA levels under MRD conditions may be challenging. It is conceivable that including a second time point would increase the sensitivity further. Although the germline filtering algorithm is accurate, it does not predict somatic origin with 100% efficiency, which could lead to a few false positives. Furthermore, limited variants detected in tissue (for example, in tumors with low TMB) cause fewer variants to be tracked, which could decrease sensitivity. Finally, this analysis was not designed to enable comparison of this new approach to alternate MRD technologies, a question that may be pursued in follow-on investigations (6).

In conclusion, this study demonstrates that monitoring variants can be derived from a tissue CGP assay without germline sampling, and postoperative ctDNA status is a strong prognostic and predictive biomarker that correlates with DFS and OS in patients with MIBC after curative-intent surgery. Early information from ctDNA could inform postsurgical clinical care by helping stratify patients who might be candidates for adjuvant treatment.

## Data availability statement

The datasets presented in this article are not readily available because we do not have IRB approval or patient consent to share individualized patient genomic data, which contains potentially identifying or sensitive patient information. Requests to access the datasets should be directed to the Foundation Medicine Data Governance Council at data.governance.council@foundationmedicine.com.

## Ethics statement

Protocol approval was obtained from independent review boards or ethics committees at each site. Informed written consent was obtained from all patients. The patients/participants provided their written informed consent to participate in this study.

## Author contributions

TP provided supervision, writing (review), and editing. AY provided supervision, conceptualization, methodology, writing (review), and editing. HN provided supervision, conceptualization, writing (review), and editing. RM provided formal analysis, data curation, visualization, writing (review), and editing. AF provided formal analysis, data curation, visualization, writing (review), and editing. DZ provided formal analysis, writing (review), and editing. YH provided formal analysis, writing (review), and editing. CX provided supervision, formal analysis, visualization, writing (review), and editing. OG provided supervision, project administration, writing (original draft and review), and editing. VA provided resources, writing (review), and editing. HW provided resources, writing (review), and editing. AA provided resources, writing (review), and editing. CC provided resources, writing (review), and editing. ND provided resources, writing (review), and editing. VD provided resources, writing (review), and editing. PG provided resources, writing (review), and editing. SM provided resources, writing (review), and editing. ES provided resources, writing (review), and editing. ZA provided resources, writing (review), and editing. GO provided supervision, conceptualization, writing (review), and editing. PH provided supervision, conceptualization, writing (review), and editing. All authors contributed to the article and approved the submitted version.
