# Supplementary material for: Molecular residual disease detection in resected, muscle-invasive urothelial cancer with a tissue-based comprehensive genomic profiling–informed personalized monitoring assay
Source: Front Oncol. 2023 Jul 31;13:1221718. doi: 10.3389/fonc.2023.1221718 (PMC10433150; doi:10.3389/fonc.2023.1221718)
Supplement: Supplementary file 1 [file DataSheet_1.docx]

Supplementary Material

Molecular residual disease detection in resected, muscle-invasive urothelial cancer with a tissue-based comprehensive genomic profiling–informed personalized monitoring assay

Thomas Powles^*^, Amanda Young, Halla Nimeiri, Russell W. Madison, Alexander Fine, Daniel R. Zollinger, Yanmei Huang, Chang Xu, Ole V. Gjoerup, Vasily N. Aushev, Hsin-Ta Wu, Alexey Aleshin, Corey Carter, Nicole Davarpanah, Viraj Degaonkar, Pratyush Gupta, Sanjeev Mariathasan, Erica Schleifman, Zoe June Assaf, Geoffrey Oxnard, and Priti S. Hegde

*** Correspondence:** Thomas Powles: thomas.powles1@nhs.net

# 1 Supplementary Figures and Tables

# 1.1 Supplementary Figures

**Supplementary Figure 1.** Comparison of the ITT population^a^ (**A** and **B**) with BEP (**C** and **D**): DFS (**A** and **C**) and OS (**B** and **D**).^b^ ^a^ITT survival curves from Powles T, et al. *Nature* 2021;595(7867):432-437. ^b^HR for ITT population is based on Cox proportional hazards model stratified by tumor stage, nodal status, and PD-L1 status. However, HR for BEP is stratified by nodal status and PD-L1 status only, owing to small strata size with tumor stage. Atezo, atezolizumab; BEP, biomarker-evaluable population; ctDNA, circulating tumor DNA; DFS, disease-free survival; ITT, intention-to-treat; Obs, observation; OS, overall survival; PD-L1, programmed cell death-ligand 1.


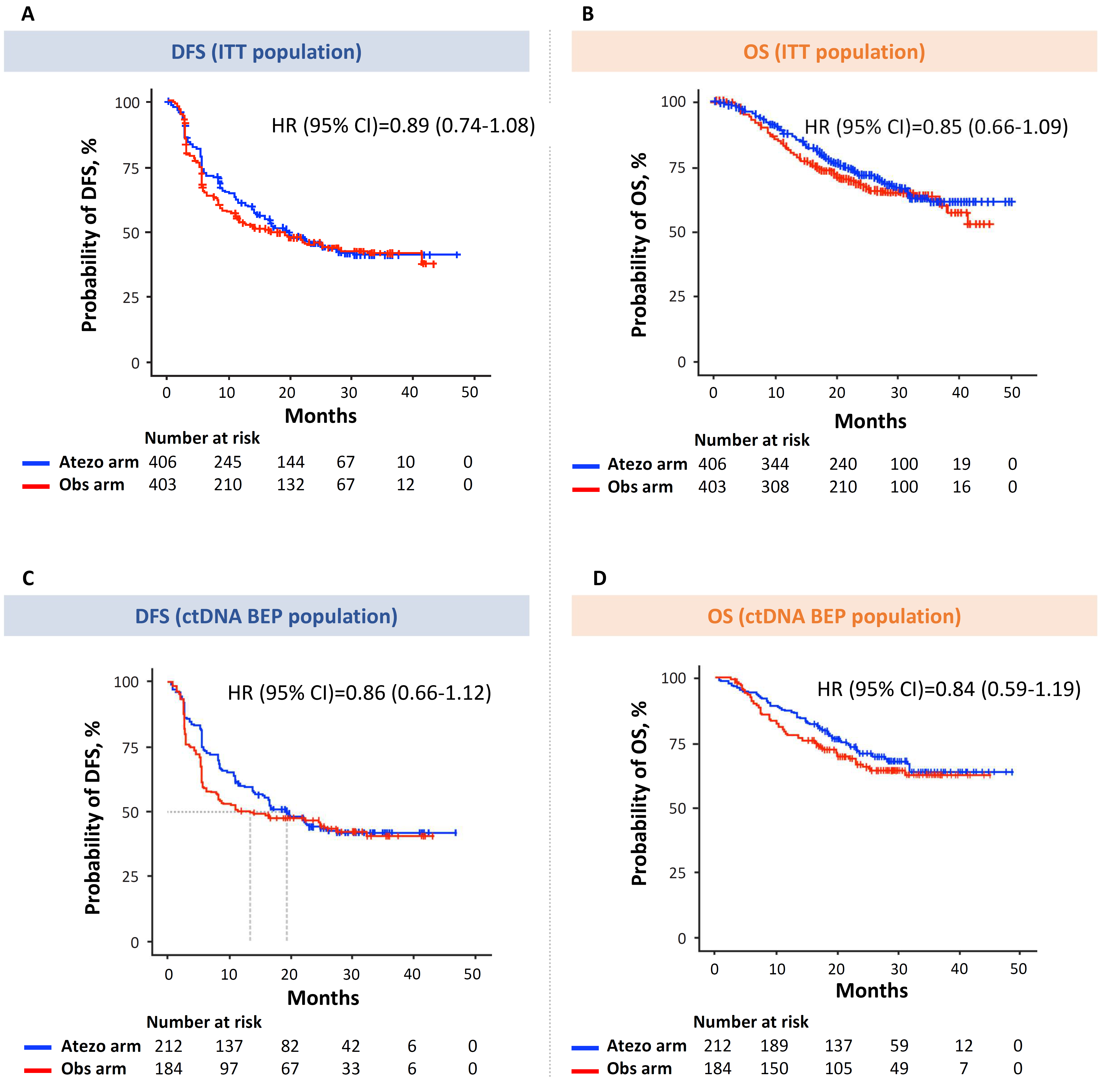


**Supplementary Figure 2.** Distribution of MTM/mL in ctDNA-positive samples by arm. ctDNA, circulating tumor DNA; MTM/mL, mean tumor molecules per mL of plasma.


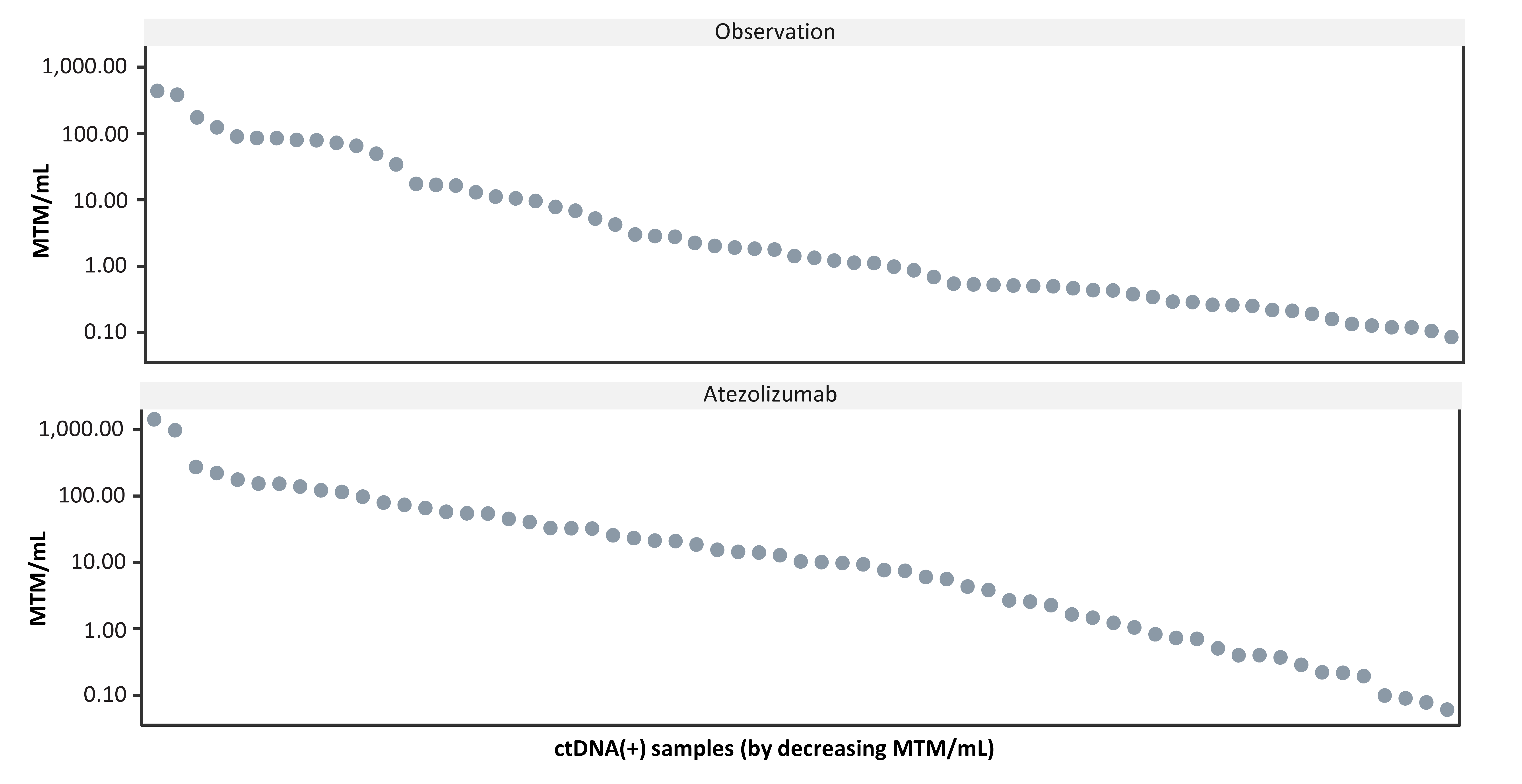


**Supplementary Figure 3.** DFS analysis by TMB in ctDNA-positive patients of the IMvigor010 study. High TMB was defined as ≥10 mutations/Mb. Low TMB was defined as <10 mutations/Mb. CI, confidence interval; ctDNA, circulating tumor DNA; DFS, disease-free survival; IC, immune cell; Mb, megabase; PD-L1, programmed cell death-ligand 1; pT, primary tumor; TMB, tumor mutational burden; tx, therapy.


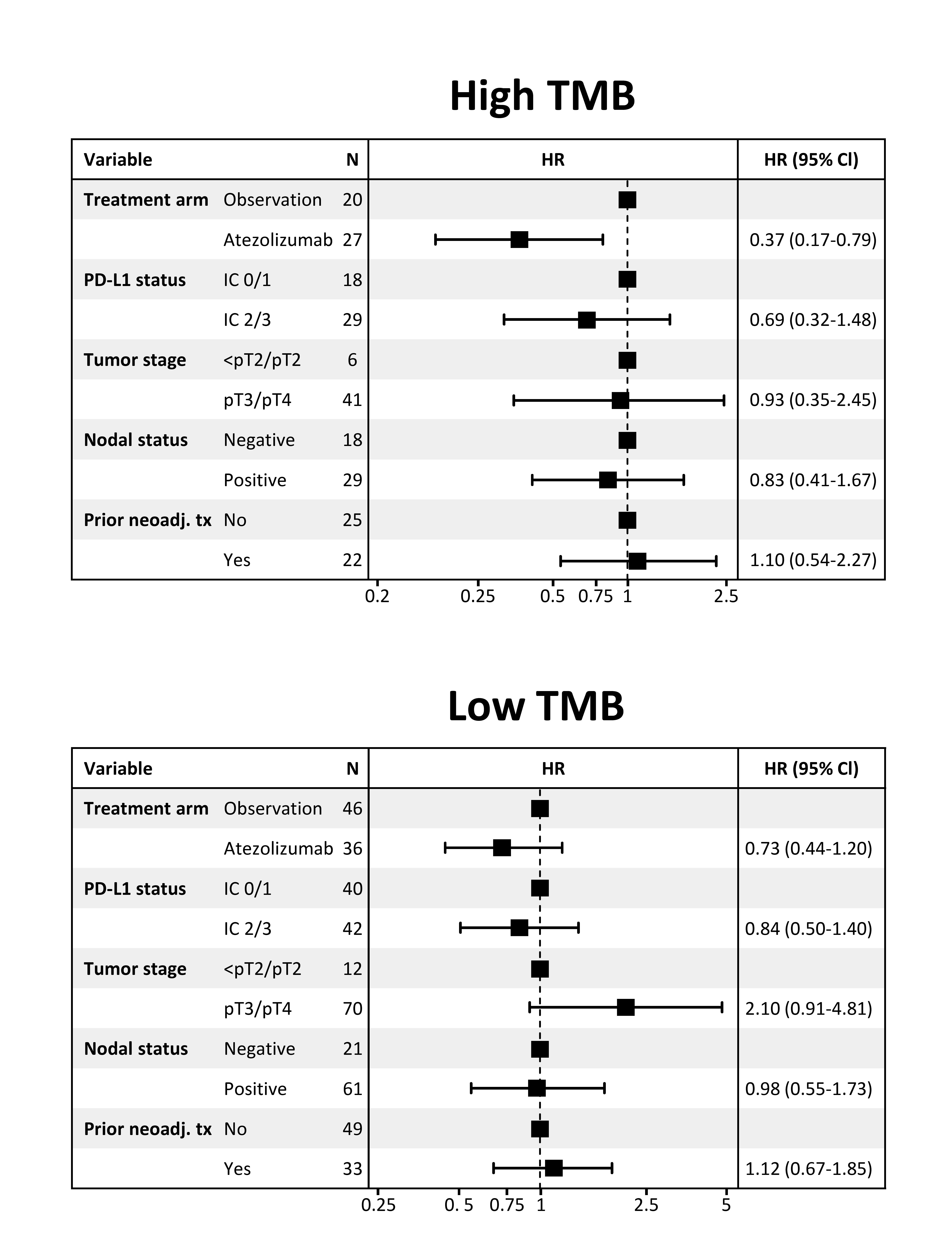


**Supplementary Figure 4.** OS analysis by TMB in ctDNA-positive patients of the IMvigor010 study. Kaplan-Meier estimates of OS for ctDNA-positive patients monitored and stratified by TMB. High TMB was defined as ≥10 mutations/Mb. Low TMB was defined as <10 mutations/Mb. ^a^Univariable. CI, confidence interval; ctDNA, circulating tumor DNA; IC, immune cell; IQR, interquartile range; Mb, megabase; NE, not evaluable; neoadj., neoadjuvant; OS, overall survival; PD-L1, programmed cell death-ligand 1; pT, primary tumor; ref, reference; TMB, tumor mutational burden; tx, therapy.





**Supplementary Figure 5.** DFS analysis by PD-L1 status in ctDNA-positive patients of the IMvigor010 study. Kaplan-Meier estimates of DFS for ctDNA-positive patients monitored and stratified by PD-L1 status (IC 0/1 vs IC 2/3). ^a^Univariable. CI, confidence interval; ctDNA, circulating tumor DNA; DFS, disease-free survival; IC, immune cell; IQR, interquartile range; neoadj., neoadjuvant; PD-L1, programmed cell death-ligand 1; pT, primary tumor; ref, reference; tx, therapy.





**Supplementary Figure 6.** OS analysis by PD-L1 status in ctDNA-positive patients of the IMvigor010 study. Kaplan-Meier estimates of OS for ctDNA-positive patients monitored and stratified by PD-L1 status (IC 0/1 vs IC 2/3). ^a^Univariable. CI, confidence interval; ctDNA, circulating tumor DNA; IC, immune cell; IQR, interquartile range; NE, not evaluable; neoadj., neoadjuvant; OS, overall survival; PD-L1, programmed cell death-ligand 1; pT, primary tumor; ref, reference; tx, therapy.





**Supplementary Figure 7.** Univariable exploratory biomarker analyses of DFS (**A**) and OS (**B**) in ctDNA-positive patients, comparing atezolizumab to observation. CDKN2A, cyclin dependent kinase inhibitor 2A; CI, confidence interval; ctDNA, circulating tumor DNA; DFS, disease-free survival; ERBB2, v-erb-b2 avian erythroblastic leukemia viral oncogene homolog 2; FGFR, fibroblast growth factor receptor; HRD, homologous recombination deficiency; MDM2, mouse double minute 2; PD-L1, programmed cell death-ligand 1; PIK3CA, phosphatidylinositol-4,5-bisphosphate 3-kinase, catalytic subunit alpha; OS, overall survival; TMB, tumor mutational burden.


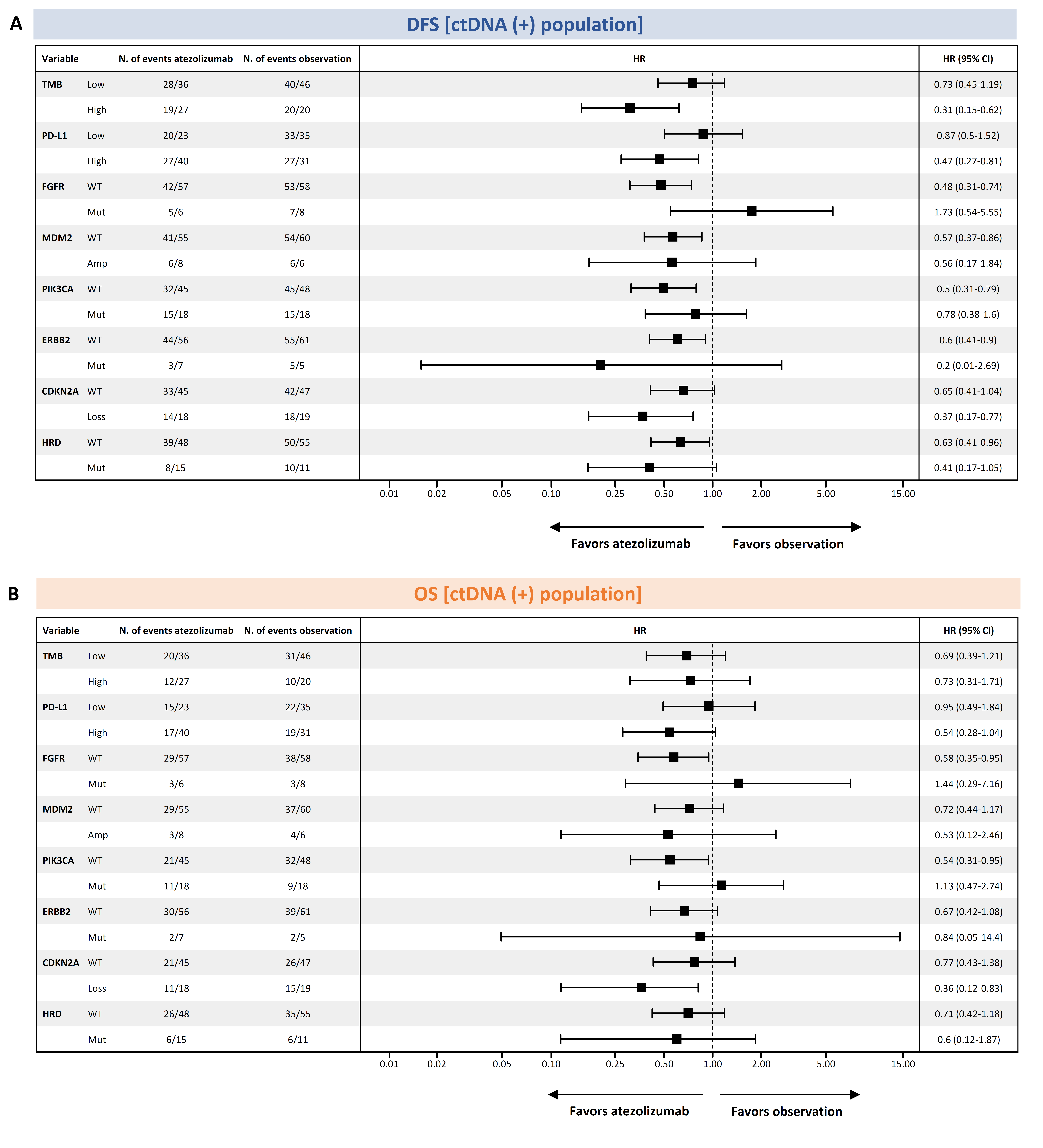


**Supplementary Figure 8.** Multivariable exploratory biomarker analyses of DFS (**A**) and OS (**B**) in ctDNA-positive patients, comparing atezolizumab to observation. CDKN2A, cyclin dependent kinase inhibitor 2A; CI, confidence interval; ctDNA, circulating tumor DNA; DFS, disease-free survival; ERBB2, v-erb-b2 avian erythroblastic leukemia viral oncogene homolog 2; FGFR, fibroblast growth factor receptor; HRD, homologous recombination deficiency; MDM2, mouse double minute 2; PD-L1, programmed cell death-ligand 1; PIK3CA, phosphatidylinositol-4,5-bisphosphate 3-kinase, catalytic subunit alpha; OS, overall survival; TMB, tumor mutational burden.


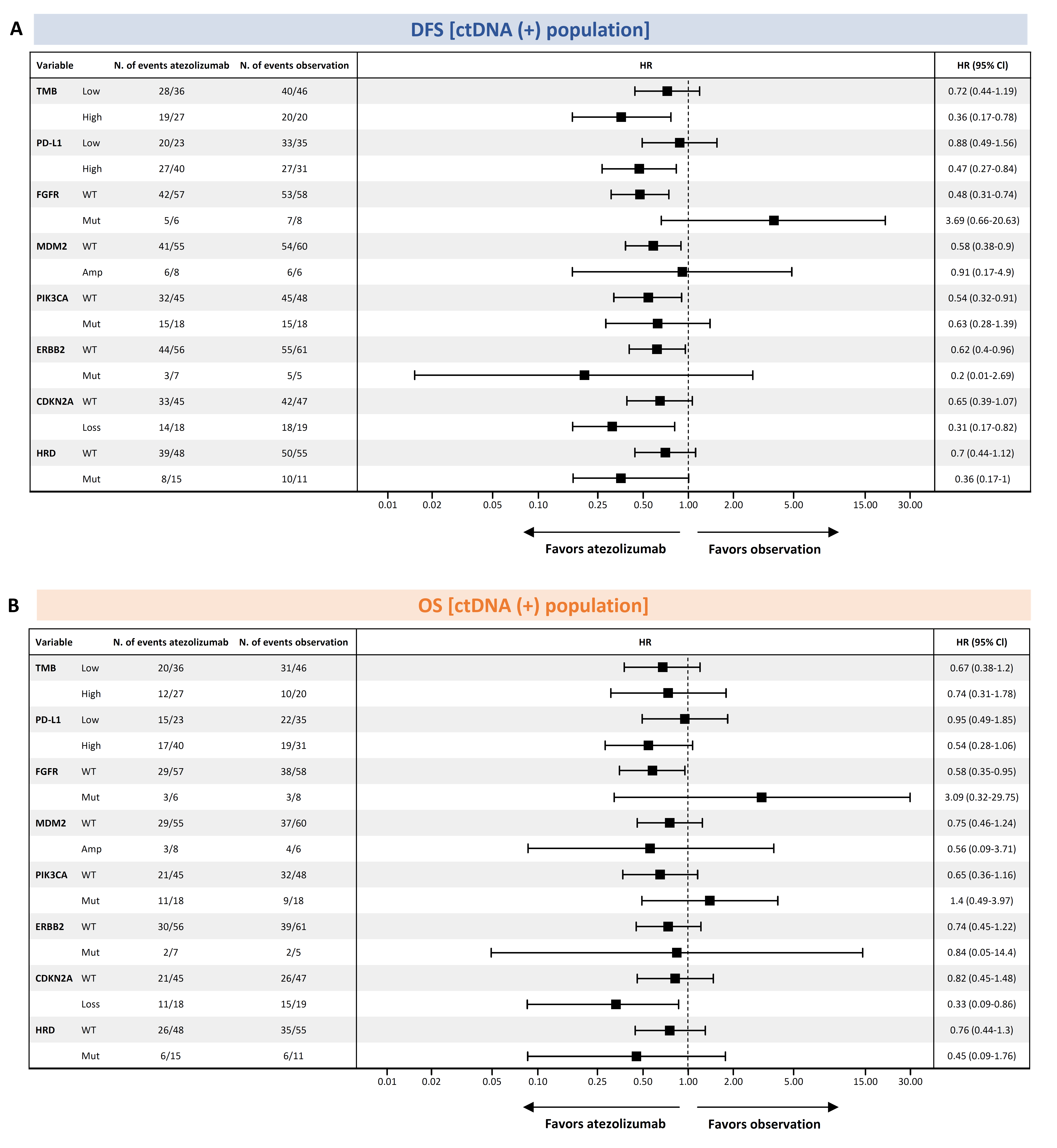


**Supplementary Figure 9.** Univariable exploratory analyses of clinical factors associated with (**A**) DFS and (**B**) OS in the observation arm. CI, confidence interval; ctDNA, circulating tumor DNA; DFS, disease-free survival; IC, immune cell; neoadj., neoadjuvant; PD-L1, programmed cell death-ligand 1; pT, primary tumor; OS, overall survival; TMB, tumor mutational burden; tx, therapy.


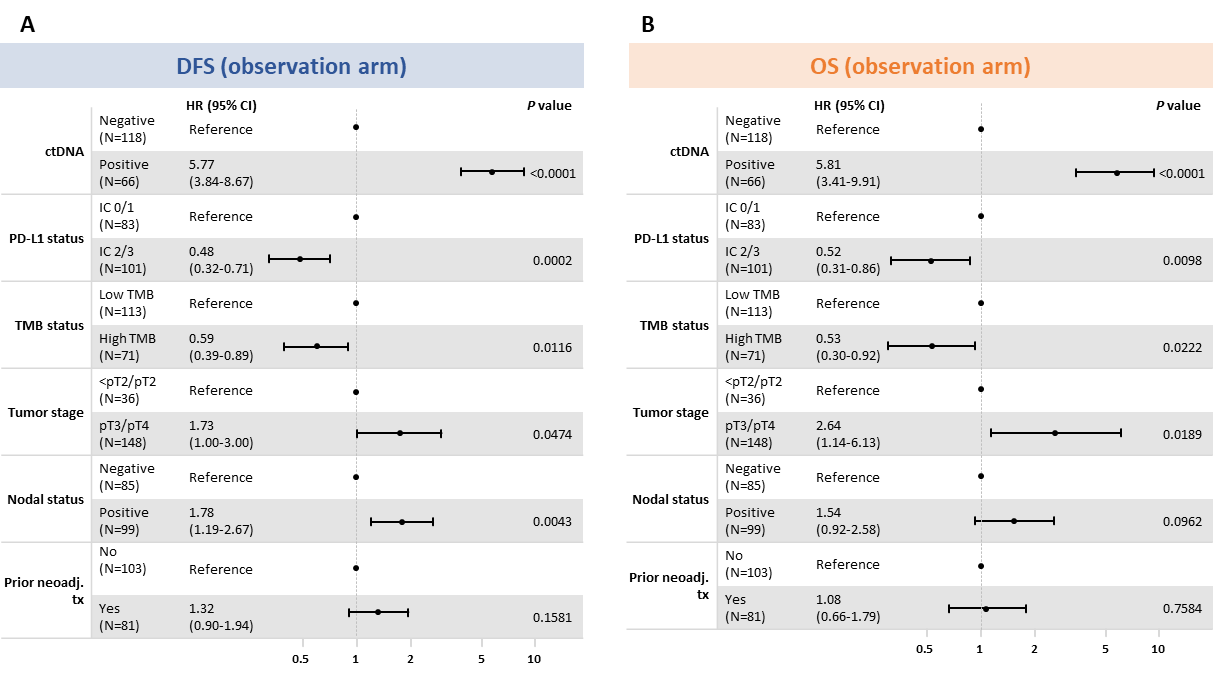


# 1.2 Supplementary tables

**Supplementary Table 1. Comparison of clinical variables in the ITT and ctDNA BEPs**

|  | **ITT population** | | **BEP** | |
| --- | --- | --- | --- | --- |
| **Clinical variable, n (%)** | **Atezolizumab**  **(n=406)** | **Observation**  **(n=403)** | **Atezolizumab**  **(n=212)** | **Observation**  **(n=184)** |
| **Median age (IQR), years** | 67 (60-72) | 66 (60-73) | 66 (59.75-72) | 67 (61-74) |
| **Gender** |  |  |  |  |
| Male | 322 (79.3) | 316 (78.4) | 158 (74.5) | 145 (78.8) |
| Female | 84 (20.7) | 87 (21.6) | 54 (25.5) | 39 (21.2) |
| **Tumor stage** |  |  |  |  |
| <pT2/pT2 | 104 (25.6) | 101 (25.1) | 38 (17.9) | 36 (19.6) |
| pT3/pT4 | 302 (74.4) | 302 (74.9) | 174 (82.1) | 148 (80.4) |
| **Nodal status** |  |  |  |  |
| Negative | 194 (47.8) | 195 (48.4) | 109 (51.4) | 85 (46.2) |
| Positive | 212 (52.2) | 208 (51.6) | 103 (48.6) | 99 (53.8) |
| **Prior neoadjuvant or adjuvant treatment** |  |  |  |  |
| No | 210 (51.7) | 214 (53.1) | 120 (56.6) | 103 (56.0) |
| Yes | 196 (48.3) | 189 (46.9) | 92 (43.4) | 81 (44.0) |
| **ECOG performance status** |  |  |  |  |
| 0 | 248 (61.1) | 259 (64.3) | 132 (62.3) | 119 (64.7) |
| 1 | 142 (35.0) | 130 (32.3) | 73 (34.4) | 57 (31.0) |
| 2 | 16 (3.9) | 14 (3.5) | 9 (4.2) | 8 (4.3) |
| **PD-L1 status** |  |  |  |  |
| IC 0/1 | 210 (51.7) | 207 (51.4) | 102 (48.1) | 83 (45.1) |
| IC 2/3 | 196 (48.3) | 196 (48.6) | 110 (51.9) | 101 (54.9) |

BEP, biomarker-evaluable population; ctDNA, circulating tumor DNA; ECOG, Eastern Cooperative Oncology Group; IC, immune cells; IQR, interquartile range; ITT, intention-to-treat; PD-L1, programmed cell death ligand 1; pT, primary tumor.

**Supplementary Table 2. DFS and OS analyses of patients monitored and stratified by ctDNA detection (MRD) at the postsurgical time point**

| **DFS** | **ctDNA (−)** | | **ctDNA (+)** | | **Observation arm (n=184)** | |
| --- | --- | --- | --- | --- | --- | --- |
|  | **Observation [ref]** | **Atezolizumab** | **Observation [ref]** | **Atezolizumab** | **ctDNA (−) [ref]** | **ctDNA (+)** |
| **Patient with events** | 43/118 (36%) | 69/149 (46%) | 60/66 (91%) | 47/63 (75%) | 43/118 (36%) | 60/66 (91%) |
| **Median DFS (95% CI), months** | NR | NR | 3.0 (2.9-5.5) | 5.9 (5.6-13.9) | NR | 3.0 (2.9-5.5) |
| **HR (95% CI)^a^** | 1.28 (0.88-1.88) | | 0.56 (0.38-0.83) | | 5.77 (3.84-8.67) | |
| ***P* value** | 0.195 | | 0.003 | | <0.0001 | |
| **OS** | **ctDNA (−)** | | **ctDNA (+)** | | **Observation arm (n=184)** | |
|  | **Observation [ref]** | **Atezolizumab** | **Observation [ref]** | **Atezolizumab** | **ctDNA (−) [ref]** | **ctDNA (+)** |
| **Patient with events** | 21/118 (18%) | 33/149 (22%) | 41/66 (62%) | 32/63 (51%) | 21/118 (18%) | 41/66 (62%) |
| **Median OS (95% CI), months** | NR | NR | 14.1 (10.5-23.0) | 23.1 (18.4-NE) | NR | 14.1 (10.5-23.0) |
| **HR (95% CI)^a^** | 1.25 (0.72-2.15) | | 0.66 (0.42-1.05) | | 5.81 (3.41-9.91) | |
| ***P* value** | 0.432 | | 0.081 | | <0.0001 | |

^a^Univariable. CI, confidence interval; ctDNA, circulating tumor DNA; DFS, disease-free survival; MRD, molecular residual disease; NE, not evaluable; NR, not reached; OS, overall survival; ref, reference.

**Supplementary Table 3. DFS analysis by TMB in ctDNA-positive patients of the IMvigor010 study**

| **DFS** | **High TMB** | | **Low TMB** | |
| --- | --- | --- | --- | --- |
|  | **Observation [ref]** | **Atezolizumab** | **Observation [ref]** | **Atezolizumab** |
| **Patients with events** | 20/20 (100%) | 19/27 (70%) | 40/46 (87%) | 28/36 (78%) |
| **Median DFS (IQR), months** | 3.8 (2.8-7.9) | 13.9 (5.6-NE) | 3.0 (2.9-5.5) | 5.6 (2.9-12.6) |
| **HR (95% CI)^a^** | 0.31 (0.15-0.62) | | 0.73 (0.45-1.19) | |
| ***P* value** | 0.00055 | | 0.21 | |

High TMB was defined as ≥10 mutations/Mb. Low TMB was defined as <10 mutations/Mb. ^a^Univariable. CI, confidence interval; ctDNA, circulating tumor DNA; DFS, disease-free survival; IQR, interquartile range; Mb, megabase; NE, not evaluable; ref, reference; TMB, tumor mutational burden.
